# Modeling protective action decision-making in earthquakes by using explainable machine learning and video data

**DOI:** 10.1038/s41598-024-55584-7

**Published:** 2024-03-05

**Authors:** Xiaojian Zhang, Xilei Zhao, Dare Baldwin, Sara McBride, Josephine Bellizzi, Elizabeth S. Cochran, Nicholas Luco, Matthew Wood, Thomas J. Cova

**Affiliations:** 1 Department of Civil and Coastal Engineering, University of Florida, Gainesville, FL 32611 USA; 2 Department of Psychology/Clark Honors College, University of Oregon, Eugene, OR 97405 USA; 3 U.S. Geological Survey, Earthquake Science Center, Moffett Field, CA 94040 USA; 4 U.S. Geological Survey, Earthquake Science Center, Pasadena, CA 91106 USA; 5 U.S. Geological Survey, Geologic Hazards Science Center, Golden, CO 80401 USA; 6 Department of Geography, University of Utah, Salt Lake City, UT 84112 USA

**Keywords:** Natural hazards, Civil engineering

## Abstract

Earthquakes pose substantial threats to communities worldwide. Understanding how people respond to the fast-changing environment during earthquakes is crucial for reducing risks and saving lives. This study aims to study people’s protective action decision-making in earthquakes by leveraging explainable machine learning and video data. Specifically, this study first collected real-world CCTV footage and video postings from social media platforms, and then identified and annotated changes in the environment and people’s behavioral responses during the M7.1 2018 Anchorage earthquake. By using the fully annotated video data, we applied XGBoost, a widely-used machine learning method, to model and forecast people’s protective actions (e.g., *drop and cover*, *hold on*, and *evacuate*) during the earthquake. Then, explainable machine learning techniques were used to reveal the complex, nonlinear relationships between different factors and people’s choices of protective actions. Modeling results confirm that social and environmental cues played critical roles in affecting the probability of different protective actions. Certain factors, such as the earthquake shaking intensity and number of people shown in the environment, displayed evident nonlinear relationships with the probability of choosing to *evacuate*. These findings can help emergency managers and policymakers design more effective protective action recommendations during earthquakes.

## Introduction

Earthquakes threaten communities across the United States. Due to the sudden-onset nature of earthquakes, it is critical to understand how people respond to earthquake hazards to recommend appropriate protective actions to save lives. Using the Protective Action Decision Model (PADM) proposed by Lindell and Perry (2012)^1^, previous studies have investigated how environmental cues, social cues, information perceptions, individual characteristics, and warning messages impact people’s protective action decision-making processes during earthquakes^2–4^. However, we argue that major research gaps remain.

The first major research gap is the modeling framework used to investigate decision-making during earthquakes. Previous studies have investigated how different factors (e.g., environmental cues and social cues) affect protective action decision-making using statistical models such as random utility models (e.g., logit model)^2,5,6^. These models usually have a predetermined (linear/log-linear) model structure. Most of these models can only identify linear trends between each factor and the target variable and are often less accurate and less flexible^7,8^. To better capture people’s dynamic behavioral responses during earthquake emergencies, we require more advanced models that can account for more complex, nonlinear relationships^9^. With the recent development of Artificial Intelligence, researchers have started to apply machine learning to model human behavior^7,9^. With more flexible model structures, machine learning models can produce highly accurate predictions and automatically identify complex relationships (e.g., nonlinearities and interactions) between decision-making behavior and different factors. However, to our knowledge, few studies have applied machine learning to investigate how different factors shape the protective action decision-making process during earthquakes^10^.

The second major research gap is the availability and use of various data types to fully understand decision-making during earthquakes. Previous studies have adopted survey and interview data when modeling the protective action decision-making process^2,11,12^. However, such data may not accurately capture the length of time required to take action, which may drive an individual’s behavioral responses^9,13^. More importantly, surveys or interviews alone cannot explicitly reflect the influence of environmental and social cues and often contain retrospective bias^14^, which limits the ability to accurately model behavior. An alternative approach is to use empirical data collected from videos such as Closed Circuit Television (CCTV) footage and phone recordings, which have unique advantages. For example, CCTV videos can detect environmental changes in the background at a given location and relate that to the impact of an earthquake^15^. More recently, with the increasing popularity of social media platforms, video data from these platforms have been leveraged to conduct protective action modeling^16^. Although video data contain rich information about people’s behavioral changes and surrounding environment, extracting information from video data is not trivial. It requires rigorous annotations and data recoding to transform the unstructured data into structured data. To our knowledge, studies using empirical data generated from CCTV footage and social media videos are still very scarce^17,18^.

To fill these knowledge gaps, this paper leverages state-of-the-art explainable machine learning methods combined with video data to model and analyze individuals’ protective actions during the earthquake. We first collected CCTV footage and videos from social media for the 2018 Anchorage earthquake. Then, we systematically annotated each individual’s time-stamped protective action decisions during the earthquake along with environmental cues such as the building settings, cover availability, and others. Also, we identified certain social cues, e.g., the role of each decision-maker and the number of people shown in the environment during the earthquake. By using the annotated data, we used a popular machine learning model, i.e., XGBoost^19^, to model the protective action decision-making process. Consequently, we applied two explainable machine learning tools, i.e., variable importance^20^ and partial dependence plots (PDPs)^20^, to interpret the model. Specifically, variable importance was used to investigate the predictive contribution of each variable, while PDPs were used to reveal their (nonlinear) relationships with the probability of choosing different protective actions at each time stage during the earthquake. Overall, the presented model and the corresponding behavioral interpretations can provide nuanced evidence for formulating more effective and context-targeted protective action recommendations.

## Literature review

### Influencing factors of protective action decision-making

Recently, several theoretical frameworks have been proposed by disaster professionals to study human behavior under emergent threats such as earthquakes and wildfires and provide guidelines to understand when and why people take action in different scenarios. One of the most widely applied frameworks is the Protective Action Decision Model^1,11^. The PADM is a multistage conceptual model that highlights people’s thinking and decision-making process in response to environmental hazards and disasters^21^. It indicates the critical characteristics to be accounted for, at both external (e.g., environmental and social cues) and internal (e.g., individual characteristics) levels. Another important theoretical framework is Emergent Norm Theory (ENT)^22,23^. ENT seeks to explain how the collective (e.g., group or crowd) behavior forms during times of crisis or uncertainty. It also highlights the importance of social and environmental cues in influencing how people respond to disasters^23^.

Social cues including social interactions, one’s proximity to others, and family concerns can affect decisions of protective actions. Studies showed that evacuees display grouping behavior, often moving with familiar individuals or authority figures and assisting those nearby^9,17^. However, the evacuation group size is negatively associated with the probability of evacuation and taking protective actions^6,24^. Also, factors that relate to family concerns such as the presence of children, are found to positively impact the likelihood of taking protective actions^11,25,26^. Overall, when multiple people are present in a room, their behavioral responses are expected to be diverse, due to differing professional capacities and social roles (e.g., parents, staff)^25^.

Environmental cues also significantly influence people’s protective action decision-making. For example, the damage status of a building^27^, the distance to exits and the flow of evacuees (i.e., number of people per second)^5^, the presence of obstacles and alarms are both found to be important components of protective action determinants^6,9,27^. Physical context factors including the ongoing activities and positions (e.g., standing, sitting, and walking), whether in a public place or private place, whether in an indoor setting or an open area also potentially affect decision maker’s choices towards protective purposes^11,25,28^.

Earthquake intensity can have a strong effect on people’s protective decision-making^17^. Researchers have suggested that people may pause and remain still for a while to receive, perceive, and assess risks, instead of immediately taking protective actions^25,29^. Higher shaking intensity may lead to less pausing and waiting time before taking protective actions^30^. In addition, aligned with our intuition, severe shaking can highly influence people’s emotional reactions and the possibility of taking protective actions^11^. Therefore, it is also necessary to consider earthquake intensity when forecasting an individual’s protective action decision-making.

Temporal features such as warning time and the time from noticing the first cue (e.g., an alert or event) are also important in influencing an individual’s behavioral responses toward natural hazards^9,16,31,32^. Other features including preparedness, prior experiences of earthquakes, risk perceptions and warning information sources are also shown to be important in determining protective actions^12,33^. In addition, demographic factors, including age, gender, education level, income, etc, are also influential to protective action decision-making^11,26,34,35^. Previous studies found that male, younger adults, more educated people and people with a relatively higher income are more likely to take protective actions during earthquakes^11,34,36^. However, regarding the specific protective actions (drop, cover and hold on, or evacuate), research has shown conflicting results^12,16,26^. For example, Shapira et al.^12^ found that people with a higher income were more likely to evacuate from buildings while Lindell et al.^11^ indicated that high-income populations were more likely to drop and find covers. We believe that these discrepancies could be mainly attributed to the differences in culture and earthquake features^12,16^. Despite the significance, these psychological and demographic features are usually collected by surveys or interviews and are not easily accessible from the footage or videos^18^.

### Approaches to modeling and interpreting protective action decision-making

The multifaceted nature of the factors influencing protective action decision-making indicates a need for flexible approaches to explore the human behavior behind the data. In recent years, based on theoretical frameworks like PADM and ENT, scholars have widely applied traditional statistical models and machine learning to model and interpret protective action decision-making^5,6,9,37^.

There exist two major categories of modeling approaches: statistical models and machine learning. Discrete choice models^38^, e.g., mixed logit and multinomial logit models, are commonly-used statistical models in exploring an individual’s decision-making process in emergencies. Built upon random utility theory, these models can quantify how individuals react to influencing factors^39^. However, these models are less accurate and less flexible since they usually have a pre-specified (linear/log-linear) model structure. Also, most of them assume that the relationship between each explanatory variable and the outcome is linear. However, the influence of environmental and social cues on an individual’s decision-making process was found to be nonlinear^9^. Accordingly, the reliability of the modeling results could be significantly impacted if we ignore the nonlinearity behind the data.

Machine learning has shown great potential in exploring the complex (e.g., nonlinear) relationships and interactions between exploratory variables, due to its flexible model structure and high prediction accuracy. Recently, several scholars have applied machine learning approaches to model protective action behavior^9,40–42^. These sets of evidence collectively suggest the capability of machine learning to model complex human behavior before, during, and after a disastrous event. To date, studies using machine learning to model protective action behavior during earthquakes are still uncommon. However, this approach is valuable because it can reveal complex, non-obvious connections between various factors and how they affect individuals’ choices during such events. Understanding these intricate relationships can greatly benefit stakeholders such as emergency managers to effectively design context-targeted solutions for earthquake protective action decision-making.

## Methods and data

### Methodology

#### Methodological framework

**Figure 1:**
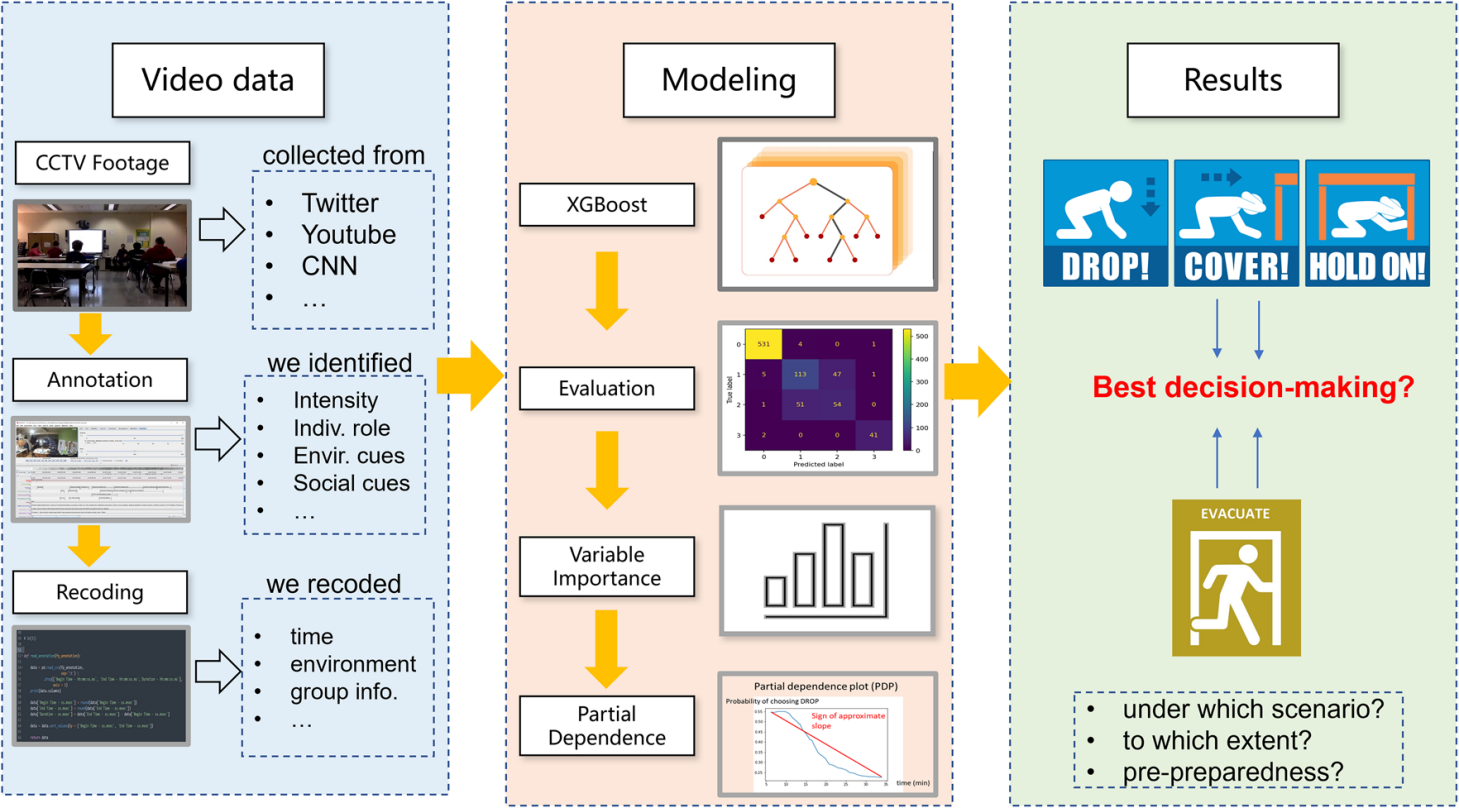
Methodological framework.

We present a methodological framework for identifying behavioral insights from earthquake protective-action decision-making. A schematic of the framework is shown in Fig. 1. We first collected CCTV footage data and personal videos posted on social media from multiple sources^43^ . After data collection, we manually annotated the data using ELAN (European Distributed Corpora Project [EUDICO] Linguistic Annotator)^44^. Specifically, we identified several key factors including the environmental cues such as alarm availability and shaking intensity, social cues such as roles of different decision-makers and leaders among the evacuation group, and most importantly, the behavior states of each decision-maker through time. We open-sourced the videos and annotations on an Open Science Framework (OSF) repository^45^ (https://osf.io/pbyzx). We transformed the annotations into numeric variables (as shown in Table S1) using *Python*^46^. We fed the data into the XGBoost model^19^ and evaluated the predictive performance using several metrics. Afterward, we interpreted the model by calculating variable importance and generating partial dependence plots^20^. The modeling results were then synthesized to draw insights about the effects of different factors on protective action decision-making. We also open-sourced the code along with an example dataset and an annotation demo (from ELAN interface) on GitHub (https://github.com/Xiaojian-Zhang/EQ_PA_ML_VideoData).

#### XGBoost model

We adopt a commonly-used tree-structured machine learning model, i.e., XGBoost^19^, to model earthquake protective action decision-making. Essentially, XGBoost relies on a process called boosting, where multiple decision trees are built sequentially, with each new decision tree correcting errors made by previous trees^19^. The boosting process ensures the improvement of predictive performance and can be operated in parallel. Therefore, XGBoost can efficiently handle large and complex datasets while maintaining high prediction accuracy, and has been widely used in behavior modeling^47,48^. In this study, suppose we use a decision tree as the base learner for XGBoost and we have training data ${\mathscr {D}} = \{{\textbf{z}}_{i} = ({\textbf{x}}_{i}, y_{i}), i = 1,2, \ldots , n\}$, with ${\textbf{x}}$ representing the input features (e.g., environmental and social cues) and *y* representing the behavioral status (i.e., *drop and cover*, *hold on*, and *evacuate*). Suppose the trees are built on *m* dimensions of features and *K* additive functions, then the XGBoost model can be formulated as:

1 $$\begin{aligned} \widehat{y}_{i} = \hat{f}({\textbf{x}}_{i}) =\sum _{k=1}^{K} f_{k}\left( {\textbf{x}}_{i}\right) , \quad f_{k} \in {\mathscr {F}} \end{aligned}$$

where in Eq. (1), ${\mathscr {F}}=\left\{ f({\textbf{x}})=w_{q({\textbf{x}})}\right\} \left( q: {\mathbb {R}}^{m} \rightarrow T, w \in {\mathbb {R}}^{T}\right)$ represents the space of all classification decision trees; *q* refers to the structure (e.g., depth) of each tree and *T* is the number of leaves in each tree. $f_{k}$ represents an individual tree constructed with structure *q* and leaf weights *w*.

#### Explainable machine learning methods

To identify the determinants of protective action decision-making and their associations, we further interpret the XGBoost model with two state-of-the-art explanation tools: variable importance and partial dependence plot^49^.

*Variable importance*. The variable importance provides the relative contribution of a variable to the predictive power of the modeling results. The more a model relies on a variable to make predictions, the more important it is. For the XGBoost model, there are three specific approaches to compute variable importance^19^. The first approach is the Gain method, which is the contribution of each feature to each tree in the model. The principle idea behind the Gain method is that by adding a new split to a variable, the two newly-generated branches will be more accurate^19,50^. Essentially, if a variable exhibits a higher Gain value relative to others, it implies that this variable plays a more crucial role in producing precise predictions. The second one is Coverage, which is measured by the relative number of observations that are related to each variable^19^. The third one is the Frequency method, which evaluates, as a percentage, the relative number of times a particular variable appears (in splits) in the trees of the model^19^. The latter two importance metrics cannot directly measure the contribution of a specific feature on improving the model’s predictive performance, and could be biased towards categorical variables. Therefore, in this study, we choose to use the Gain method.

*Partial dependence plot*. While variable importance is comparable to the magnitude of the beta coefficient in traditional statistical models such as multinomial logit (MNL) model, it cannot indicate the direction (i.e., sign) of the variable’s relative contribution. PDPs reveal the direction of associations between the predictors and the target variable^49^. Introduced by Friedman^51^, PDP can display the marginal effect that the studied variable has over the predicted outcome^20^. Specifically, suppose the set *S* contains the features of interest and set *C* is the complement of *S* and let ${\textbf{x}}$ denote the predictors, the PDP works by marginalizing the predicted outcome over $\mathbf {{x_{C}}}$. Eq. (2) defines the partial dependence of the model *f* on $\mathbf {{x_{S}}}$:

2 $$\begin{aligned} \hat{f}_\mathbf {{x_{S}}}\left( \mathbf {{x_{S}}}\right) =E_{\mathbf {{X_{C}}}}\left[ \hat{f}\left( \mathbf {{x_{S}}}, \mathbf {{x_{C}}} \right) \right] \end{aligned}$$

In this study, $\hat{f}_{\mathbf {{x_{S}}}}$ is estimated by taking the average of the data samples, as shown in Eq. (3) where *n* is the number of total instances:

3 $$\begin{aligned} \hat{f}_{\mathbf {{x_{S}}}}\left( \mathbf {{x_{S}}}\right) =\frac{1}{n} \sum _{i=1}^{n} \hat{f}\left( \mathbf {{x_{S}}}, \mathbf {{x_{C}}}^{(i)} \right) \end{aligned}$$

With PDP, we are able to investigate the average marginal effect of a given value of feature(s) in *S* on the protective action decision-making.

#### Model training and performance evaluation

We split the entire dataset into two disjoint sets, 90% for training purposes and the remaining 10% for testing purposes. We adopted a stratified sampling technique to preserve the distribution of target variable in each split. We tuned the XGBoost with Grid Search and a five-fold Cross Validation on the entire training set^52^. The grid search space is described as follows. We examined the effect of the number of trees by varying the number from 100 to 700 at an interval of 100; we tested the learning rate using values ranging from 0.01 to 0.05 with a step of 0.01; we also evaluated the depth of each individual by setting the range as 4 to 6. Consequently, the XGBoost was built with 100 trees with a learning rate of 0.05. Each tree has a depth of 5. After obtaining the best hyperparameter combination, we evaluate the model’s predictive performance on the testing set.

Several performance metrics were used to evaluate the model’s predictive performance. These metrics include accuracy, recall, precision and F1 score^52^. Accuracy reflects the model’s predictive accuracy in correctly predicting the observed protective action decisions. Recall measures the proportion of correctly predicted instances in a behavioral state against the total instances of that state. Precision is the ratio of correct predictions for a specific behavioral state to the total number of predictions made for that behavioral state. F1 score is the harmonic mean of recall and precision, providing a balance between them.

### Performance comparison

This study compares the performance of XGBoost with a commonly-used traditional statistical model, i.e., MNL^53^. MNL is based on a random *utility* maximization framework, where it assumes that each choice (i.e., protective action) offers each individual (i.e., decision-maker) a specific level of utility. MNL consists of two major parts: (1) the effects of the observed variables and (2) a random error term that is independently and identically Gumbel-distributed^53^. Mathematically, the utility of choosing protective action *j* can be written in Eq. (4):

4 $$\begin{aligned} U_j\left( {\textbf{x}}_j \mid \mathbf {\beta }_j\right) =\mathbf {\beta }_j^T {\textbf{x}}_j+\mathbf {\varepsilon }_j, \end{aligned}$$

where $\mathbf {\beta }_j$ is the coefficient vector of the corresponding ${\textbf{x}}_j$ and $\mathbf {\varepsilon }_j$ is the unobserved random error when choosing protective action *j*. The probability of choosing protective action *j* for decision-maker *i* can be formulated as Eq. (5):

5 $$\begin{aligned} p_{i j}=\frac{\exp \left( \mathbf {\beta }_j^T {\textbf{x}}_{i j}\right) }{\sum _{p=1}^m \exp \left( \mathbf {\beta }_p^T {\textbf{x}}_{i j}\right) } \end{aligned}$$

Given the coefficients, the MNL can then be formulated by the likelihood function in Eq. (6):

6 $$\begin{aligned} {\varvec{L}}(\mathbf {\beta })=\prod _{i=1}^N \prod _{j=1}^J\left[ \frac{\exp \left( \mathbf {\beta }_j^T {\textbf{x}}_{i j}\right) }{\sum _{p=1}^m \exp \left( \mathbf {\beta }_p^T {\textbf{x}}_{i j}\right) }\right] \end{aligned}$$

We can adopt maximum likelihood estimation technique to estimate the coefficients $\hat{\mathbf {\beta }}=\arg \max _{\mathbf {\beta }} {\varvec{L}}(\mathbf {\beta })$. And then we can substitute $\hat{\mathbf {\beta }}$ back to the Eq. (5) to obtain the probability of choosing each protective action.

### Data

#### Background of 2018 M7.1 anchorage earthquake

**Figure 2:**
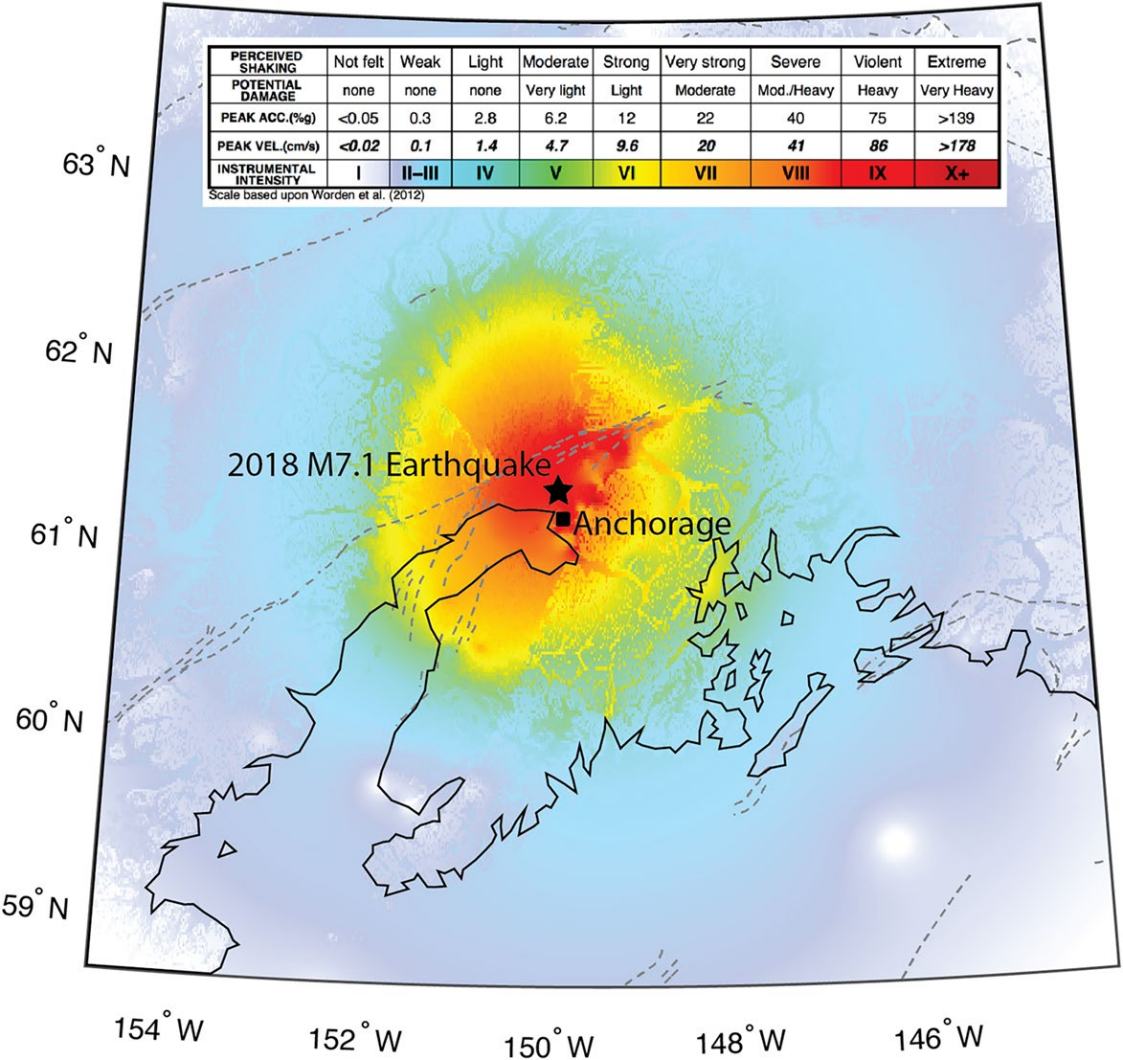
Distribution of shaking intensities near the 2018 M7.1 Anchorage earthquake as reported by the U.S. Geological Survey^55^. The epicenter of the earthquake (black star) was located just to the north of the population center of Anchorage (black square). Coastlines are shown by black lines and major fault traces are shown by the dashed gray lines.

The 2018 M7.1 Anchorage earthquake occurred on the morning of November 30, 2018 at 8:29 am local time^54^. The earthquake started about 10 km north of the city of Anchorage at a depth of almost 50 km, occurring within the Pacific Plate that is being subducted beneath the North American plate^54^. The resulting shaking was the strongest to impact Anchorage in more than 50 years. Shaking intensities across the city of Anchorage were Modified Mercalli Intensity (MMI) VII–VIII (very strong to severe), as reported by residents and recorded on seismometers^55^. The distribution of shaking intensities is shown in Fig. 2. Shaking, and secondary impacts such as ground failure, resulted in damage to many buildings including hospitals, schools, and lifeline infrastructure such as water and power lines^54,55^.

#### Data collection

After the Anchorage 2018 earthquake, a Virtual Emergency Response Team (VERT) was initiated to identify and download publicly available video footage of the earthquake that had been voluntarily posted to social media websites, please refer to McBride et al. (2022)^43^ for the sources of the videos. A total of 90 videos depicting Anchorage-related earthquake footage were identified. Only 66 of these displayed unique content, and of these, only 45 displayed human behavior during earthquake shaking. 37 videos displayed behavior inside a structure (25 were public structures and 12 were private structures). Children were present in 43% of the 45 human behavior videos, and 60% of those 45 videos depicted CCTV footage (the other 40% depicted footage filmed from a handheld camera or phone). Further detail about the VERT process for identifying the videos can be found in McBride et al. (2022)^43^. Note that this project has received an exemption determination from the University of Oregon (UO) Institutional Review Board (IRB) (#10302019.043) for using publicly available, earthquake-related videos from social media. We strictly follow the privacy guidelines and ensure our research remains within the scope of the exemption.

#### Data annotation and recoding

Open-source ELAN (European Distributed Corpora Project [EUDICO] Linguistic Annotator) software^44^ was utilized to support the annotation of the video footage. A multi-tier template for annotation was designed specifically for the purpose of annotating earthquake-related behavior. The template guided the time-locked transcription of unfolding earthquake-related events and people’s behavior, including all interpretable action, language, gesture, and emotion produced during the videos. The lexicon also included codes related to demographic variables of interest, such as inferred age, gender, ability status, and nature of relationship to others present (e.g., parent, child, teacher, student, security guard, member of general public, etc.). An estimate of the number of people present was also provided, and a judgment was made about the extent to which the setting was crowded depending on the number of people shown in the environment, where they are, the density of people, and the crowdedness of the physical environment such as furniture quantity and arrangement. A lexicon of earthquake events (e.g., shaking begins and ends, initiation of P-wave in audio signal, etc.), earthquake-related events that might trigger human action (e.g., alarm sounds, observable shaking commences, objects sway, objects fall, obstacles present on the floor, etc.), protective actions (e.g., run, evacuate, drop, cover, hold on, etc.), social responses (e.g., announce earthquake, alert others, direct others, etc.), and whether an alarm sounded (e.g., building fire alarm, car alarm, etc.) was also created to guide annotation. Judgments of shaking intensity were also provided. To ensure the reliability of the annotations, we have undertaken a formal reliability analysis for some aspects of the annotation process with regard to coder-annotators’ judgments that involved the lexicon. The inter-annotator reliability estimate yielded an overall percent agreement of 94%. Please refer to Supplementary Material for more details.

In this study, we adopted a multi-phase annotation procedure. Specifically, in the first phase, all 45 videos with evident human behavior during earthquake received full annotations of these above-mentioned characteristics. As this study focused on the individual-level behavior, annotators then provided a judgment of whether or not a leader/decision-maker was present within the video (e.g., a parent, teacher, security guard, etc.) and whether or not a follower was present (e.g., a child, a student, member of the general public, etc.). Annotations were then standardized and conducted, for any given video, on the individual level for both one leader/decision-maker and one or two followers depicted within each video. It is worth noting that, in many videos, others were also present in addition to the individuals identified as leaders or followers. However, the data presented here are based solely on the behavior of the leaders and followers. Here, we treat each individual, no matter whether a leader or follower, as a decision-maker. Lastly, 17 videos received the final phase of annotation and were fully annotated with leader and follower designation. These videos were then utilized for follow-up analysis. For a detailed description and corresponding statistics, please refer to Table S1.

We translated all annotations into numeric variables, including the behavioral state, shaking intensity, whether an alarm was on, if there were obstacles present, whether the environment was crowded, availability of cover, the starting position of the decision maker, whether the decision maker was a leader, and whether the decision maker was far from the egress. All variables were calculated and recoded at each timestamp (per-second level). Recoding was conducted at the individual level (each decision maker). In total, we processed 17 videos with a total of 1593 unique behavioral statuses. The mean video length was 41 s; the standard deviation was 51 s. The longest video was around 216 s while the shortest only lasted for 10 s. On average per video, 6.6 people were present. As we described above, we only annotated some of the individuals as leader/decision-maker or followers. Accordingly, on average one leader and two followers’ behavioral states were identified per video.

#### Descriptive statistics

Table S1 summarizes all variables we include in this study and their descriptive statistics. Among the 17 annotated videos, a large majority of them (87.1%) were filmed in a public setting. About one third (32.4%) of videos displayed evidence of a leader present. Most people were sitting (74.6%) when the earthquake began and at a considerable distance from egress (67.0%). We observed nine levels of shaking intensity. “Not felt” accounted for the largest proportion and most of the remaining observations were concentrated between MMI 3 (weak shaking intensity) to MMI 6 (strong shaking intensity), although videos included shaking up to MMI 8 (severe shaking). Observations regarding whether an alarm was present were highly imbalanced with only 4.4% of observations with an alarm (not from an earthquake early warning system but a school alarm triggered after onset of an emergency). Obstacles were not present in 57.1% of observations, while 42.9% showed evidence of obstacles (e.g., shifted furniture, swing doors and falling objects). 12.9% of observations were in uncrowded environments, while 87.1% were in crowded environments. Availability of cover, such as a table, was only observed in 12.0% of videos. The target variable, i.e., the protective action behavioral state, had four categories: *other*, *drop and cover*, *hold on*, and *evacuate*. The behavioral state *other* (e.g., communicating and seeking proximity with others) accounted for more than 55% of observations, followed by *drop and cover* with 26.9%, *hold on* with 8.8%, and *evacuate* with 8.7%. The distributions shown in Table S1 and the descriptions above show a strong class-imbalance issue (i.e., certain categories are significantly more frequent than others) existing among almost all variables.

## Results

This section will first introduce the predictive performance comparison between machine learning and traditional statistical model. Then, we will present the predictive contribution for each variable using variable importance. Finally, we identify the potential nonlinear associations between each variable that has high importance values and protective action decision-making.

### Predictive performance

Table 1 displays the calculated performance metrics. The overall prediction accuracy of XGBoost is 95%, and that of MNL is 66.9%. The flexible model structure and the strength of capturing nonlinear relationships behind the data enable XGBoost to deliver highly accurate protective action predictions. In terms of classifying the behavioral status, XGBoost is highly accurate in predicting *evacuate* and *drop and cover*, followed by *hold on*. This finding holds across the results calculated by both precision, recall and F1-score. By contrast, MNL has less predictive power for predicting each protective action, especially for *hold on* (none of the predictions is correct). This result is intuitive since MNL has a fixed (less flexible) model structure, which limits its capability in capturing the complex relationships of the data.

**Table 1:** Testing-set predictive performance of MNL and XGBoost.

Protective action	Share of obs.	Precision		Recall		F1-score	
		MNL	XGBoost	MNL	XGBoost	MNL	XGBoost
Other	0.556	0.717	0.978	0.798	1.000	0.755	0.989
Drop and Cover	0.269	0.587	0.909	0.628	0.930	0.607	0.920
Hold on	0.088	0.000	0.833	0.000	0.714	0.000	0.769
Evacuate	0.087	0.600	1.000	0.643	0.929	0.621	0.963

Overall Accuracy: MNL: 0.669, XGBoost: 0.950.

### Variable importance

**Figure 3:**
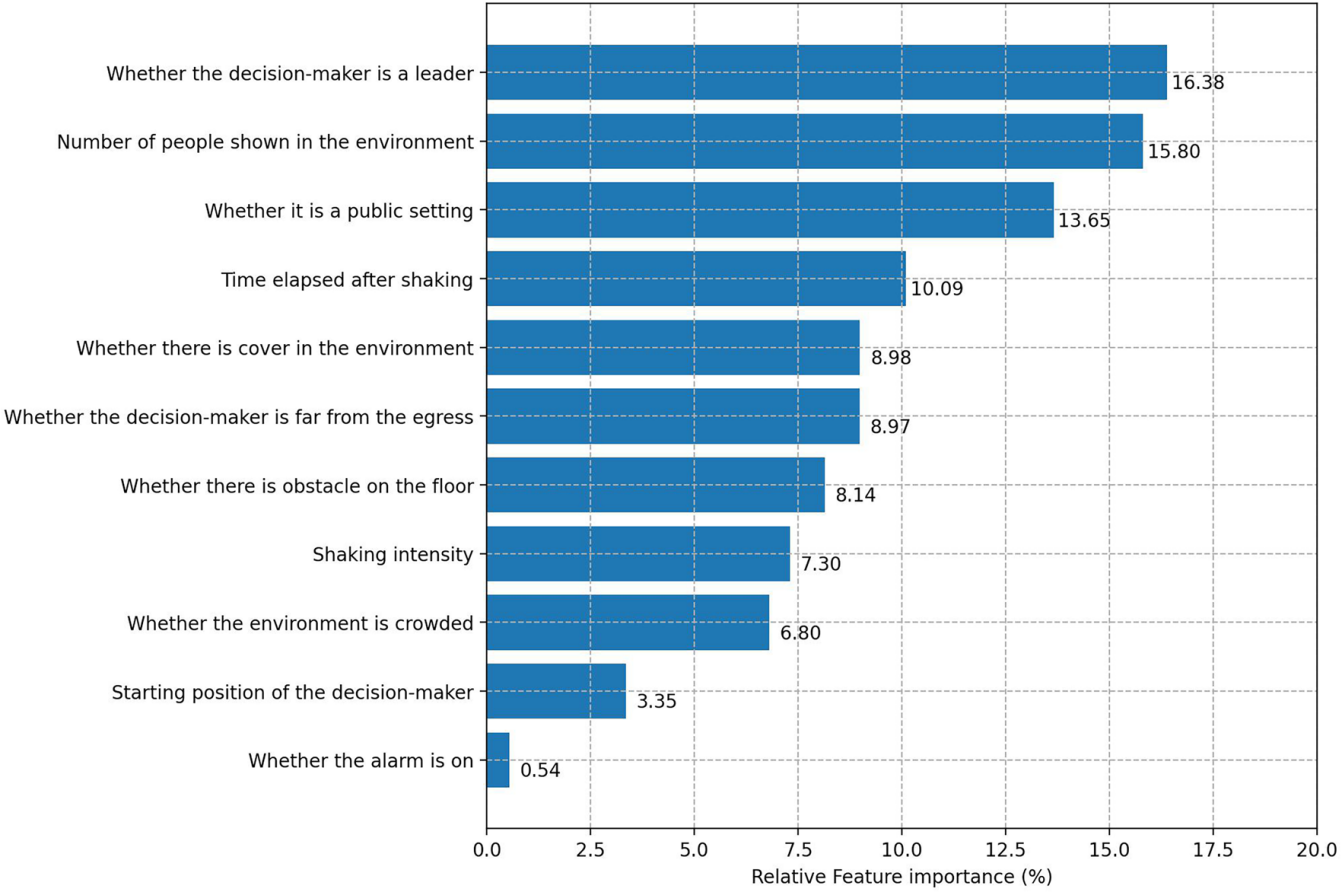
Variable importance.

Figure 3 presents the variable importance, i.e., the predictive power of each variable on classifying protective action decision-making. Results show that the leader indicator variable has the highest influence (16.38%) on the decision outcome. This suggests that social influence plays an important role in protective action decision-making^9,56^. The leader’s decision may highly impact the other’s decisions regarding how to respond to earthquake threats. The number of people in the environment accounts for the most important (15.80%) environmental cue, followed by whether it is a public setting (13.65%), whether cover is available (8.98%), whether he/she is far away from the egress (8.97%), whether there is an obstacle on the floor (8.14%), earthquake shaking intensity (7.30%), and whether the environment is crowded (6.80%). Time elapsed after the video starts (10.09%) is also of great importance in forecasting the decision-making process. The starting position (e.g., sitting versus standing) of the decision maker when an earthquake occurs only accounts for 3.35%. Finally, whether an alarm is on only shows a 0.54% predictive importance for protective action decision-making. We also calculated variable importance aggregated by categories (as shown in Table S1). Overall, time-varying variables contribute to 26.1% predictive importance whereas static variables collectively account for 73.9% predictive power. On average, each time-varying variable accounts for 6.5% importance while each static variable has 10.6% importance.

### Nonlinear relationships

**Figure 4:**
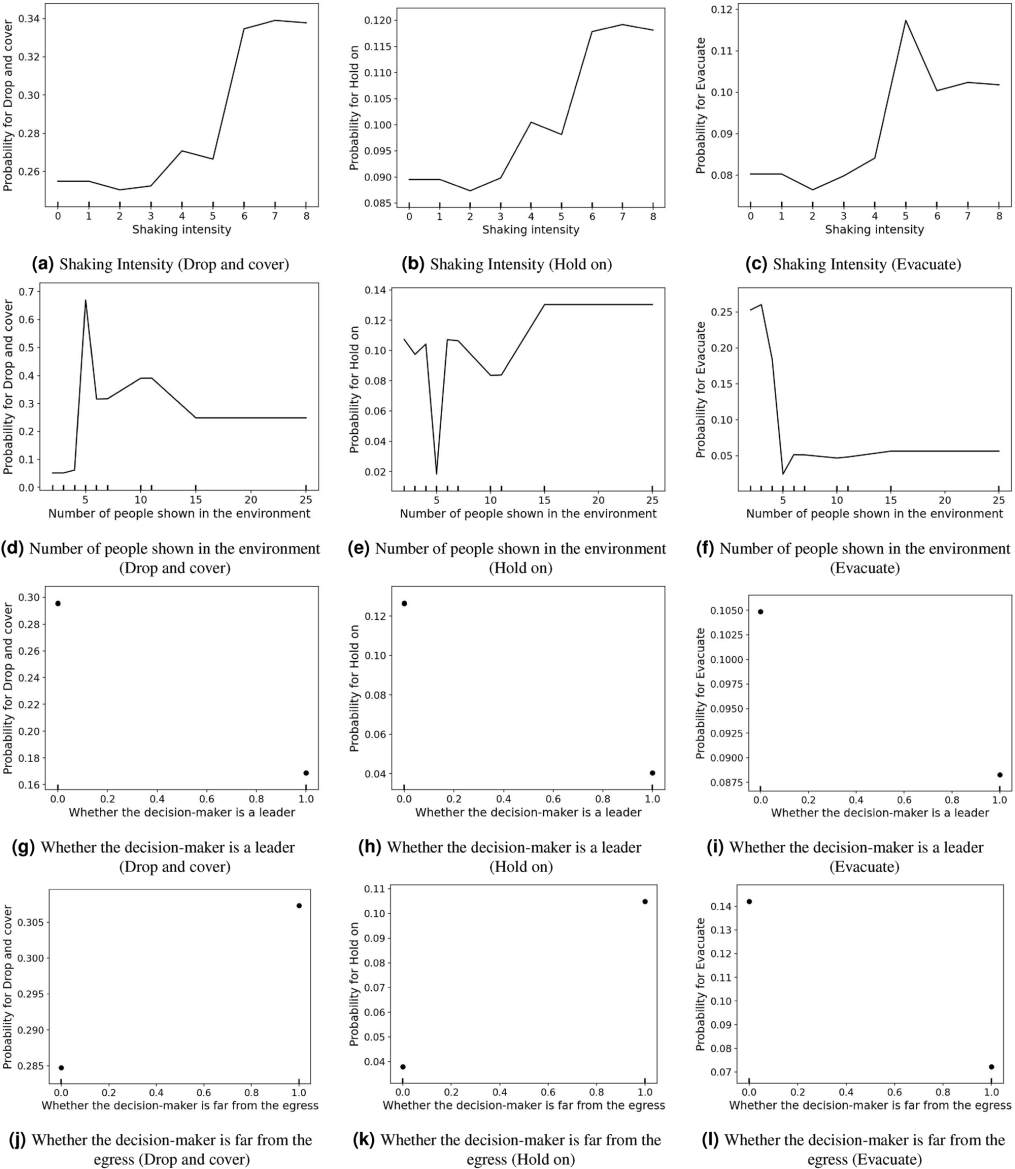
Partial dependence plots. The *y*-axis is the probability of choosing different protective actions. The *x*-axis is the value range for the variable of interest. The rug marks (i.e., tick marks) at the bottom of each plot show the distribution of the variable of interest. Note that the *y*-axis limit changes across protective actions and variables.

While the variable importance suggests variables have differential abilities to predict outcomes, it is still not possible to directly connect each variable with specific protective-action decision-making. To address this, we adopted PDP to examine their relationships. The results of PDP for time-varying and static variables for the three behavioral statuses (*drop and cover*, *hold on*, and *evacuate*) with predictive importance larger than 5% were generated. Four selected variables are illustrated in Fig. 4, including shaking intensity, the number of people, if the decision-maker is a leader, and if the decision-maker is far from egress. For other variables, please refer to Fig. S1. The rug marks (i.e., tick marks) at the bottom of each plot show the distribution of the variable of interest. Note that the reliability of the results may be compromised in regions with fewer data points (where the rug marks are sparse).

Shaking intensity is positively associated with choosing *drop and cover* and/or *hold on* (Fig. 4a,b). Similarly, as shaking intensity increases from no shaking to MMI V (particularly from MMI IV-V), people are more likely to *evacuate* (Fig. 4c). However, after the shaking intensity exceeds MMI V, such that the shaking intensity increases from moderate to strong, the probability of *evacuate* drops rapidly between MMI V-VI and then plateaus at a medium level.

The number of people shown in the environment (Fig. 4d–f) has a nonlinear relationship with protective action decision-making. Specifically, when the number of people increases from 0 to 5, the probability of choosing *drop and cover* significantly increases. However, when the number of people exceeds 5, the probability of choosing *drop and cover* gradually decreases. This finding suggests that this variable has a threshold effect, where the threshold is 5. When the number of people is less than 5, there may be more opportunities to find cover; while it is difficult for a large group of people to find available shelters/covers. A threshold effect is also shown in the PDP of *evacuate*. As the number of people rises from 0 to 5, the probability of choosing *evacuate* first slightly increases (the peak is when the number of people is 3) and then drastically drops. Above 5, the probability of *evacuate* remains flat.

The decision maker being a leader relates negatively with the likelihood that they will undertake any of the three protective actions, as shown in Fig. 4g–i. A leader is more likely to guide others (e.g., the teacher guides the students) than to take protective action(s). Figure 4j–l show that when the decision maker is far away from the egress, *drop and cover* or *hold on* are more likely, while it is less likely that the decision maker will *evacuate*.

Figure S1a–c present that time elapsed has a positive correlation with a decision-maker’s probability of choosing *evacuate*. This aligns with our intuition because as time goes on, the shaking intensity may become weaker, allowing people to evacuate from the building. By contrast, time elapsed is negatively associated with the probability of choosing *drop and cover* or *hold on*. Figure S1d–f show that the presence of obstacles on the floor has a negative relationship with the probability of *drop and cover* or *evacuate*, whereas it relates positively to the probability of hold-on actions. Figure S1g–i display that people are more likely to *hold on* and *evacuate* in a crowded environment while less likely to drop and find cover. Figure S1j–l show that cover availability is positively associated with the possibility of choosing *drop and cover* or *hold on* while it is negatively associated with *evacuate*. This finding suggests that when cover is available, the decision-maker is more likely to take a local action, rather than risking evacuating during shaking. The PDPs of the public setting indicator (Fig. S1m–o) show that a public setting has a positive association with choosing to *hold on* and *evacuate* but a negative relationship with the probability of *drop and cover*.

## Discussion

Based on the results above, in this section, we will discuss (1) the strengths of adopting explainable machine learning for modeling and interpreting protective-action decision-making, and (2) major findings with their connections to the existing literature and implications for future decision-making.

### Strengths of adopting machine learning

First, *accuracy*. Machine learning models can outperform traditional statistical models in terms of prediction accuracy^7,47,57^. The present study provides additional confirmation on this front. For example, Table 1 suggests that the machine learning model has a 95% prediction accuracy, significantly outperforming the traditional statistical model. The superior predictive performance can be attributed to machine learning’s more flexible model structure, enabling it to capture complex underlying relationships behind the data. Specifically, a pre-specified model structure is not required for machine learning models, and, therefore, it can effectively capture the interactions among variables^7^. Our machine learning model showed the highest prediction accuracy when forecasting *evacuate*, followed by *drop and cover* and *hold on*. This finding holds for all evaluation metrics including Precision, Recall and F1-score. One possible explanation for the relatively lower accuracy of *hold on* is that this behavior has an observation share of only 8.8% and is in an intermediate behavioral stage. Fewer observations and the underlying connections between *drop and cover* as well as *evacuate* may introduce significant difficulties in the prediction of *hold on* for the machine learning model. The high performance on forecasting *evacuate* is probably because evacuation usually has unique characteristics such as the decision maker being in close proximity to an exit. *Drop and cover* also showed high prediction accuracy; its recall rate is higher than precision. A lower precision rate suggests that machine learning has a comparatively higher likelihood of incorrectly classifying other protective actions (e.g., *hold on*) as *drop and cover*.

Second, *nonlinear relationships*. Another important advantage of using a machine learning model is that machine learning can automatically capture nonlinear relationships between explanatory variables and the target variable^9^. This merit enables us to identify more detailed nonlinear relationships between each feature and protective behavior. Traditional statistical models usually only offer the direction of associations, i.e., negative or positive^7^. Relationships between behaviors under investigation could display greater systematic diversity that is not captured by such models. For example, we would not expect there to be a linear (or monotonic) relationship between shaking intensity and the probability of choosing *evacuate*, given that shaking intensity changes dramatically over the course of the earthquake^17^. Using a machine learning model, we found that the effects of shaking intensity on the probability of choosing *evacuate* is nonlinear (i.e., an inverse U-shape) and there exists a threshold (i.e., MMI V), as shown in Fig. 4c. The revealed nonlinear relationships and identified thresholds can significantly enhance our understanding of how different explanatory variables shape protective actions and provide useful insights for policymakers to develop more effective protective-action guidelines.

### Major findings

Whether the decision-maker is a leader demonstrated the highest predictive importance (15.65%) among all predictors. This finding reinforces the critical role of social interactions in determining protective-action behaviors^25,34^. When focusing on the association of the leader/follower variable with each behavior, we found that decision-makers being a leader are less likely to have any protective action (e.g., *drop and cover*, *hold on*, or *evacuate*). One possible reason is that the leaders observed are mostly parents, teachers, or directors; and they tend to provide immediate guidance and reach out to individuals who may need assistance (e.g., children or students) due to responsibilities and professional capacities rather than taking immediate protective actions themselves^34^.

Distance from egress was found to be predictively significant in shaping protective action choices. This finding aligns with our intuition that a decision-maker who is close to the egress is more likely to *evacuate*, while others at a greater distance from egress may opt to drop and find cover. The partial dependence plots, as depicted in Fig. 4j–l, further confirm our intuitions. Previous studies have also reported a negative relationship between distance to exits and evacuation choices inside buildings^5,58^. However, a threshold for the distance from egress, or more fundamentally, the perception of the distance from egress that could determine whether an individual is more likely to *evacuate* or choose to *drop and cover*, and *hold on*, remains unknown. Answers to this question are critical for understanding which protective actions people are inclined to take based on their location^32^. Nevertheless, hints towards this threshold may be concealed within our dataset, as estimating the actual distance from egress from videos is challenging. Future studies may consider exploring such a threshold by integrating videos and surveys^5^ or simulations^27^.

The number of people in the environment was a strong predictor for forecasting protective actions; however, the specific relationship between the number of people and the probability of response was complex. This finding aligns with a recent study by Zhao et al.^9^: on the one hand, the authors showed that the relationship between group size and the probability of responding to an emergency, such as a fire alarm, was not monotonic. At the same time, it was observed that the presence of more individuals responding within a group tended to increase the chance of others also choosing to respond. This finding highlights the important role of social interactions in influencing an individual’s decision-making during an emergency. The probability of choosing to *evacuate* suddenly decreased when the number of people reached five and then remained almost stable. Previous studies reflected a similar finding. For example, Lovreglio et al.^6^ found that group size was negatively associated with the probability of evacuation. Additionally, people were more likely to *evacuate* from a building if they were alone^24^, perhaps because they were less likely to be influenced by others’ behavioral responses. We also found that the probability of choosing to *drop and cover* significantly increased before the number of people reached five, but with larger numbers of people, it gradually dropped. With fewer people, there are likely more opportunities to find cover. However, it might become more difficult for a larger group of people to find available cover.

Another interesting variable is shaking intensity. Our empirical results showed that shaking intensity also influences decisions around protective actions. Figure 4c shows that MMI V may be a threshold for deciding to *evacuate* or not, which addresses a research question proposed by Lambie et al.^17^. The research question pertains to whether there exists a specific level of shaking intensity that triggers particular responses. People might be able to move around and seek an exit to *evacuate* when shaking intensities are weak (e.g., less than MMI V). As shaking intensity grows larger than MMI V, the intense nature of the shaking may impede an individual’s mobility and reduce the probability of *evacuate*. However, scholars have argued that people’s behavioral responses could be diverse so that observations of certain protective actions may not be transferable globally to different contexts^29^. Therefore, further investigations could test if the observed threshold holds for other scenarios or contexts.

We should note that previous studies found that personal experience and risk perceptions also play essential roles in people’s protective action decision-making^2,12,34–36^. However, we are not able to capture such factors from each individual via the video data. Our machine learning-based approach, even excluding personal experience and psychological factors, shows promising results in forecasting the protective actions of decision-makers, providing new insights for response behaviors during earthquakes.

## Conclusion

Using CCTV footage and videos collected from the 2018 Anchorage earthquake, this study examines the application of machine learning in modeling protective action decision-making. Overall, it contributes twofold to the literature. First, we show that machine learning methods have strong capabilities for modeling protective action decision-making during earthquakes. Specifically, they can not only deliver better prediction accuracy than commonly-used traditional statistical models , but also offer broader insights into behavioral interpretations. In particular, machine learning models automatically capture the complex, nonlinear relationships between various factors (e.g., social and environmental cues) and choices for protective actions (i.e., *drop and cover*, *hold on*, *evacuate*). Second, this study is one of the first few studies that employ real-world CCTV footage and videos to investigate protective action behavior during earthquakes. The results have shown great potential for such audio-visual data to enhance understanding of human behavior under emergency scenarios. Also, the results and insights can be further used for improving the design of earthquake early-warning systems^18,59^. In short, this study can provide a foundation for emergency managers, policymakers, and safety professionals to develop more effective protective-action recommendations.

Despite the contributions, this study has limitations that open avenues for future research. We argue that this work could be conceptualized as a pilot study for several reasons. Firstly, this study only adopts 17 videos (albeit with 1593 unique behavioral statuses) for modeling. Future research could expand the video sample size by incorporating empirical data from multiple earthquake events. This would not only enhance the robustness of the results but also provide a more comprehensive understanding of protective action decision-making processes across different contexts. Secondly, while utilizing CCTV footage and video data shows great potential, these sources fall short in accurately capturing certain crucial aspects such as sociodemographic factors, prior experience, and individuals’ perceptions of risk. In fact, prior research has demonstrated that these factors are also influential in decision-making during emergencies^2,12,34^. Future studies might consider integrating multi-source data, e.g., combining surveys with CCTV footage and videos, to better understand how various factors interplay with people’s perceptions of issues like timing and understanding critical barriers to ultimately shape protective action decisions.

### Supplementary Information

Supplementary Information.

## Data Availability

Videos and annotations that support the findings of this study are published on an Open Science Framework repository^45^: https://osf.io/pbyzx.

## Code availability

The analysis was conducted using *Python*^46^. Code to reproduce our main results is available on GitHub: https://github.com/Xiaojian-Zhang/EQ_PA_ML_VideoData.
