# Supplementary material for: Modeling protective action decision-making in earthquakes by using explainable machine learning and video data
Source: Sci Rep. 2024 Mar 5;14:5480. doi: 10.1038/s41598-024-55584-7 (PMC10914816; doi:10.1038/s41598-024-55584-7)
Supplement: Supplementary file 1 — Supplementary Information. [file 41598_2024_55584_MOESM1_ESM.pdf]

Modeling Protective Action Decision-Making in Earthquakes by Using Explainable Machine Learning and Video Data

Xiaojian Zhang[1,*], Xilei Zhao[1], Dare Baldwin[2], Sara McBride[3], Josephine Bellizzi[2], Elizabeth S. Cochran[4], Nicholas Luco[5], Matthew Wood[6], Thomas J. Cova[6]
[1]Dept. of Civil and Coastal Engineering, University of Florida, Gainesville, FL 32611
[2]Dept. of Psychology/Clark Honors College, University of Oregon, Eugene, OR 97405
[3]U.S. Geological Survey, Earthquake Science Center, Moffett Field, CA 94040
[4]U.S. Geological Survey, Earthquake Science Center, Pasadena, CA 91106
[5]U.S. Geological Survey, Geologic Hazards Science Center, Golden, CO 80401
[6]Dept. of Geography, University of Utah, Salt Lake City, UT 84112
[*]Corresponding author, xiaojianzhang@ufl.edu

Supplementary Information

Methods and Data

Inter-annotator reliability test

We have undertaken a formal reliability analysis for some aspects of the annotation process with regard to coder-annotators' judgments that involved the "lexicon." In particular, a random subset of the full set of Anchorage videos has been recoded independently by a second coder-annotator, and a reliability estimate is being calculated for those judgments. The reliability estimate we conducted was based on the following strategy. Specifically, we used the annotations as the basis for coding a given whole video with respect to whether 1) any one of Participant1 (P1), P2, and/or P3 (if a P3 was present) displayed a given protective-action behavior at any point during the video, 2) an alarm sounded, 3) footage was filmed inside, and 4) footage was CCTV vs. handheld. This strategy results in a relatively small sample of observations (10 coding categories for 17 videos = 170 observations). Of those 170 observations, 160 displayed independent coder agreement, yielding an overall percent agreement of **94%**, which suggests a high degree of agreement.

Tables

Table S1. Variable description and statistics. The "Value" column represents the recoded values for each variable, while the "Description" column provides a brief description of the variable. The "Count" column indicates the number of observations with a specific value, and the "Share%" column denotes the proportion of observations with that value. Additionally, the table includes the mean and standard deviation (SD) for each variable. During data pre-processing, we utilized the variance inflation factor (VIF) to assess multicollinearity among independent variables. It's important to note that more variables were initially tested than what is shown in the table. However, two variables, namely Decision-makers' group size and a binary variable indicating the presence of a

child/children, were excluded from modeling due to high correlation with other variables. All remaining variables in the table have a VIF value below 10, suggesting that multicollinearity issues should not be of concern[1,2].

	Value	Description	Count	Share %	Mean	SD
Target Variable						
Behavioral_State		Protective action			0.7	1
	0	Other (e.g., direct others)	886	55.6		
	1	Drop and cover	429	26.9		
	2	Hold on	140	8.8		
	3	Evacuate	138	8.7		
Time-varying Variable						
Shaking_Intensity		Earthquake shaking intensity			2.9	2
	0	No shaking (MMI NA)	18	1.1		
	1	Not felt (MMI I)	631	39.6		
	2	Weak (MMI II) / Light (MMI II)	126	7.9		
	3	Weak (MMI III) / Light (MMI III)	206	12.9		
	4	Light (MMI IV)	168	10.5		
	5	Moderate (MMI V)	229	14.4		
	6	Strong (MMI VI)	182	11.4		
	7	Very srong (MMI VII)	27	1.7		
	8	Severe (MMI VIII)	6	0.4		
Time_Elapsed		Time elapsed after shaking			54.2	61.1
Alarm_On		Whether the alarm is on			0	0.2
	0	No	1523	95.6		
	1	Yes	70	4.4		
Obstacle_Floor		Whether there is obstacle on the floor			0.4	0.5
	0	No	909	57.1		
	1	Yes	684	42.9		
Static Variable						
Envir_Crowded		Whether the environment is crowded			0.1	0.3
	0	No	205	12.9		
	1	Yes	1388	87.1		
Cover_Availability		Whether there is cover in the environment			0.9	0.3
	0	No	1402	88		
	1	Yes	191	12		
Start_Pos_DM		Starting position of the decision-maker			1.6	0.7
	0	Other (e.g., unknown)	162	10.2		

	1	Standing	242	15.2		
	2	Sitting	1189	74.6		
Num_People		Number of people shown in the environment			7.1	4
Public_Setting		Whether it is a public setting (school, work, airport, etc.)			0.8	0.4
	0	No	285	17.9		
	1	Yes	1308	82.1		
DM_Leader		Whether the decision-maker is a leader			0.3	0.5
	0	No	1077	67.6		
	1	Yes	516	32.4		
DM_Far_Egress		Whether the decision-maker is far from the egress			0.7	0.5
	0	No	525	33		
	1	Yes	1068	67		

Figures

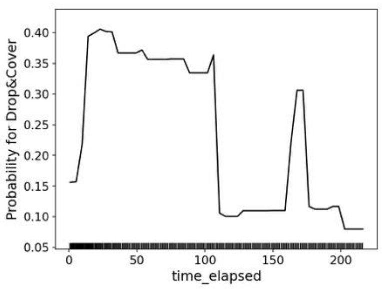

(a) Time elapsed after shaking (Drop and cover)

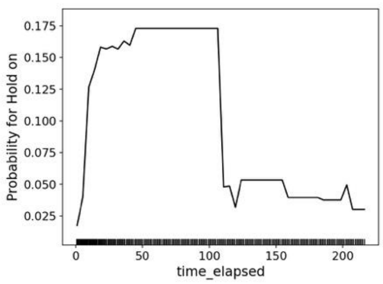

(b) Time elapsed after shaking (Hold on)

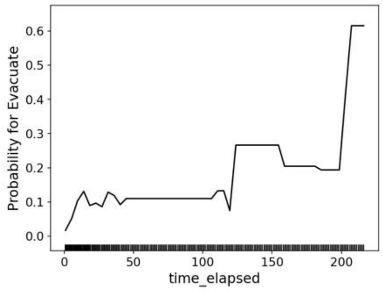

(c) Time elapsed after shaking (Evacuate)

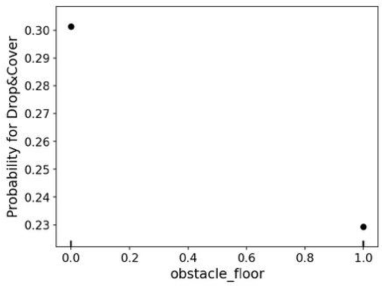

(d) Whether there is obstacle on the floor (Drop and cover)

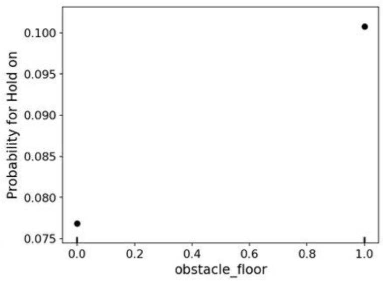

(e) Whether there is obstacle on the floor (Hold on)

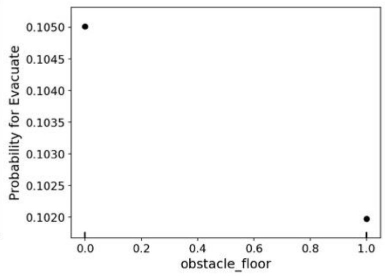

(f) Whether there is obstacle on the floor (Evacuate)

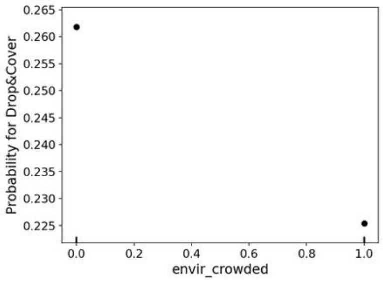

(g) Whether the environment is crowded (Drop and cover)

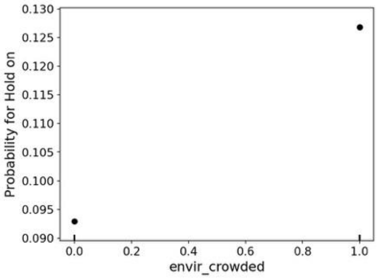

(h) Whether the environment is crowded (Hold on)

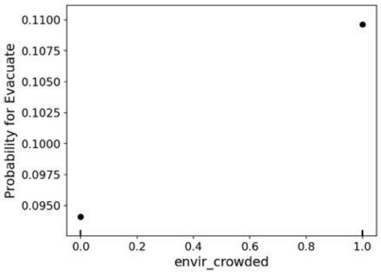

(i) Whether the environment is crowded (Evacuate)

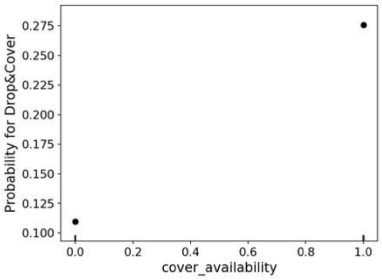

(j) Whether there is cover in the environment (Drop and cover)

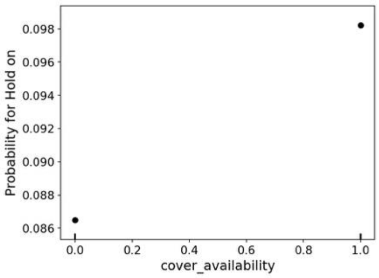

(k) Whether there is cover in the environment (Hold on)

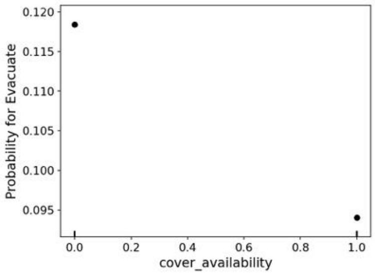

(l) Whether there is cover in the environment (Evacuate)

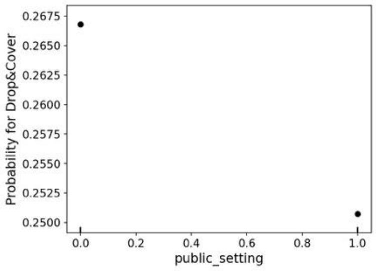

(m) Whether it is a public setting (Drop and cover)

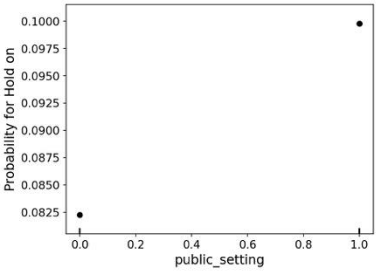

(n) Whether it is a public setting (Hold on)

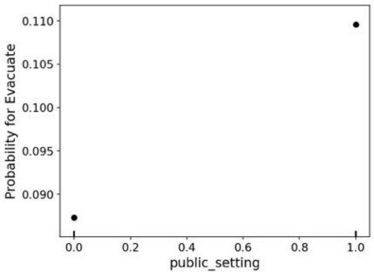

(o) Whether it is a public setting (Evacuate)

Figure S1. Partial Dependence Plots. The *y*-axis is the probability of choosing different protective actions. The *x*-axis is the value range for the variable of interest. The rug marks (i.e., tick marks) at the bottom of each plot show the distribution of the variable of interest. Note that the *y*-axis limit changes across protective actions and variables.

Data availability

Videos and annotations that support the findings of this study are published on an Open Science Framework (OSF) repository (Zhang and Baldwin, 2024): https://osf.io/pbyzx.

Code availability

The analysis was conducted using *Python* (Rossum et al., 1995). Code to reproduce our main results is available on GitHub: https://github.com/Xiaojian-Zhang/EQ_PA_ML_VideoData.

Acknowledgment

This research was supported by a United States Geological Survey (USGS) supplement award to the National Science Foundation grant (No. 1921157) as well as a USGS Intergovermental Personnel Act (IPA) to the University of Oregon. Any opinions, findings, and conclusions or recommendations expressed in this material are those of the authors and do not necessarily reflect the National Science Foundation. ShakeAlert® is a registered trademark of the (USGS) and is used with permission. Any use of trade, firm, or product names is for descriptive purposes only and does not imply endorsement by the U.S. Government.

This study has received an exemption determination from the University of Oregon (UO) Institutional Review Board (IRB) (#10302019.043) for using publicly available, earthquake-related videos from social media. We strictly follow the privacy guidelines and ensure our research remains within the scope of the exemption.

References

1. Xu, Y., Yan, X., Liu, X., Zhao, X., 2021. Identifying key factors associated with ridesplitting adoption rate and modeling their nonlinear relationships. *Transportation Research Part A: Policy and Practice* **144**, 170–188.

2. Yan, X., Liu, X., Zhao, X., 2020. Using machine learning for direct demand modeling of ridesourcing services in Chicago. *Journal of Transport Geography* **83**, 102661.

3. Zhang X., and Baldwin, D. *PA Videos and Annotations*. https://osf.io/pbyzx.

4. Van Rossum, G., Drake, F. L. et al. *Python reference manual*, vol. 111 (Centrum voor Wiskunde en Informatica Amsterdam,1995)
